# The influence of age and sex on carcass characteristics and chemical composition of the longissimus thoracis et lumborum muscle in wild boars (*Sus scrofa*)

**DOI:** 10.5194/aab-64-199-2021

**Published:** 2021-05-27

**Authors:** Tomasz Żmijewski, Monika Modzelewska-Kapituła

**Affiliations:** Department of Meat Technology and Chemistry, Faculty of Food Sciences, University of Warmia and Mazury in Olsztyn, Plac Cieszyński 1, 10-719 Olsztyn, Poland

## Abstract

The aim of this study was to determine the influence of age and sex on carcass characteristics and the chemical composition of the longissimus thoracis et lumborum (LTL) muscle in wild boars (*Sus scrofa*). Carcass quality parameters varied significantly depending on age and sex, whereas the protein and collagen contents in the muscle were affected by animal age. The carcasses of male yearlings and adults were characterised by the highest processing suitability, which can be attributed to the highest percentage of lean meat in the carcass and a moderate fat and bone content. A higher fat content was found in carcasses of females from all age groups, and a lower bone content was found in yearlings and adult females. The protein content was the highest in the LTL muscle of adult boars, and the collagen content was the highest in piglets; thus, the chemical composition of the muscle was most desirable in adult wild boars and least desirable in piglets.

## 1 Introduction

Wild boars (*Sus scrofa*) inhabit the entire European continent, excluding Ireland, England and the Scandinavian countries (small populations are found in Denmark and southern Sweden), large parts of Asia, Indonesia and North Africa. The species has also been introduced to North and South America (Sales and Kotrba, 2013). In Poland, the wild boar is one of the most numerous wild animals (after roe deer, hares and pheasants) and is also one of the most frequently hunted (Ludwiczak et al., 2019). Wild boars have steadily expanded their territory, and their population has continued to grow, mainly due to their phenotypic plasticity (Castillo-Contreras et al., 2021), ability to adapt to changing environmental conditions, high reproductive success, omnivorous nature, resistance to climatic conditions, declining populations of large predators, insufficient hunting pressure from humans and climate changes (Scillitani et al., 2010; Amici et al., 2015). Decreasing woodland area and changes in crops structure towards increasing areas devoted to corn production have also favoured the increase in wild boar numbers due to a significant increase in the availability of high-energy food, and this has resulted in wild boar overpopulation (Ludwiczak et al., 2019). Another possible impact of corn farming on the wild boar population stems from the high risk of mycotoxin (zearalenone) creation by *Fusarium* during the decomposition of corn cobs on the ground. Mycotoxin, which is delivered to wild boars via the ingestion of maize grains, results in changes in the animals' reproductive system and cycle, as it is one of the strongest non-steroidal oestrogenic substances (Popczyk, 2016). Thus, it has been suggested that mycotoxin intake might be the reason for reproductive disturbances in wild boar populations in Poland, where sows with piglets can currently be observed nearly all year round. Apart from a longer breeding season, the sows producing offspring are younger than in the past, indicating that the age of sexual maturity has been decreasing (Pałubicki et al., 2021). Other important factors influencing the size of the domestic wild boar population are African swine fever, an imbalance in the ratio between male and female population numbers, the harvesting structure utilised and the provision of fodder for animals during winter (Popczyk, 2016).

**Figure 1:**
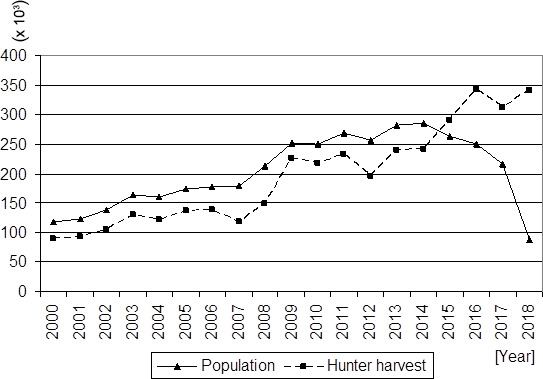
Population and hunter harvest of wild boars in Poland (own study based on the Concise Statistical Yearbook of Poland, 2020).

Poland has a large population of wild boars that has been growing steadily in recent years (Fig. 1). Over the past 18 years, the wild boar harvest rate has increased by more than 350 %, and 341 000 animals were harvested in 2018. However, a decrease in the wild boar population has been noted since 2015, and a dramatic decrease in the population was observed in 2018. This resulted from the high wild boar harvest rate and from the presence of African swine fever (ASF), the latter of which has been occurring over a growing area of Poland. The consequence of maintaining a high harvest rate and the ASF-related animal mortality was a lower wild boar population in March before the breeding period (when animals were counted) than the number of wild boars generally present during the year (Fig. 1). Additionally, the disproportionate results stem from the fact that young wild boars born after March and shot during the year are not included in the population census for a given year, but they are included in the harvest records.

Due to their dietary preferences, wild boars cause a significant damage to agricultural crops, and their population has to be controlled by hunting (Herrero et al., 2006; Amici et al., 2012). Before ASF, the rules for managing wild boar populations in Poland were strictly defined: consistent hunting of piglets, reduced hunting of yearlings and limited harvest of older animals to 10 % of the total harvest, and no hunting of 3- to 4-year-old wild boars (Kujawskie Koło Łowieckie, 2012). Based on the recommended harvest structure, which included 60 % piglets (< 1 year), 30 % yearlings (1–2 years) and 10 % older animals (Przybylski, 2006), the average carcass weight was estimated to be 40 kg. Despite the relatively low carcass weight, more than 13 500 t of wild boar meat was harvested annually in Poland. Due to the treatment of ASF, the rules have become less strict, and hunters are currently permitted to harvest animals of any sex or age to 250 % of total spring population (Main Board of Polish Hunting Association, 2020). Therefore, with a higher harvest of older wild boars, the amount of meat obtained annually may be even greater. Generally, the species has a high economic importance, and it is the largest source of game meat in Poland. According to a review article by Sales and Kotrba (2013), wild boar meat and processed products have been insufficiently researched and described. Published data indicate that environmental (age, body weight, body condition score and health status, habitat, food availability and season) and genetic factors influence carcass traits (Skewes et al., 2008; Żochowska-Kujawska et al., 2010b; Tesarova et al., 2018) as well as the chemical composition and properties of wild boar meat (Żochowska et al., 2005; Żochowska-Kujawska et al., 2010a; Dannenberger et al., 2013; Pedrazzoli et al., 2017; Russo et al., 2017). However, studies carried out to date have not included the determination of the yield of primal cuts, the tissue composition of the carcass and primal cuts, and the classes of meat obtained from wild boar of different ages and sexes. The loin (m. longissimus thoracis et lumborum, LTL) is one of the most valuable cuts from a wild boar carcass and is highly appreciated in gastronomy. During the animal's life, the muscle plays a supportive function and may therefore be less affected by animal age than locomotive muscles (e.g. semitendinosus or semimembranosus). The LTL muscle is also frequently used in studies focused on the quality of wild boar meat. Because of the shortage of information on the effects of wild boars' sex and age on the loin quality, we chose the LTL muscle as the focus of this study. Due to the fact that age and sex are the key determinants of carcass and meat quality, the aim of this study was to determine their influence on carcass characteristics. The hypothesis that animal age and sex would affect the quality of the wild boars loin was also tested.

## 2 Materials and methods

### 2.1 Animals

The wild boars (*Sus scrofa*, n=48) used in this study were harvested by hunters in winter (10 hunts in 10 subsequent weeks) in north-eastern Poland. The animals were shot during a regular hunt (collective hunting without dogs) conducted according to Polish hunting law (Hunt Law, Polish Law Journal No. 147/713, 1995; see the Regulation of the Minister of the Environment of 22 March 1995 for detailed conditions regarding the hunting and marking of carcasses, given in Polish Law Journal No. 61/548, 2005). Due to the fact that the carcasses used in the study were obtained from animals harvested during hunts, the study did not require approval from an ethics committee. The animals were shot in ASF-free areas. They were eviscerated on the hunting ground and chilled to a temperature below 7 ${}^{\circ }$C, in line with the hygiene procedures for harvesting game (Regulation (EC) No 853/2004, 2004; Tropiło and Kiszczak, 2008). After 72 h chilling, carcasses were obtained by removing the skin from the distal segments of forelimbs at the carpal joint, hind limbs at the tarsal joint and the head at the atlanto-occipital joint, and they were divided into primal cuts. A carcass was defined as the body of a harvested wild boar without abdominal and thoracic organs, urogenital organs, visceral fat, esophagus, larynx, trachea, tongue, skin, tail, distal limb segments and the head (Standard BN-84/924/-10, 1984).

Carcasses were divided into three groups based on animal age (piglets, yearlings and adults). Each group was further subdivided into sexes to produce a total of six groups that each consisted of eight carcasses. The following groups were identified: group I 

 – male piglets (males aged <1 year), group I 

 – female piglets (females aged <1 year), group II 

 – male yearlings (males aged 1–2 years), group II 

 – female yearlings (females aged 1–2 years), group III 

 – adult males (males aged 2–3 years), group III 

 – adult females (females aged 2–3 years). The age was estimated based on the growth and replacement of the teeth.

### 2.2 Carcass characteristics

The following carcass traits were analysed: the weight and percentage of primal cuts; tissue composition – the content of lean meat, bones and fat in primal cuts and in the carcass; and the division of meat into quality classes.

**Figure 2:**
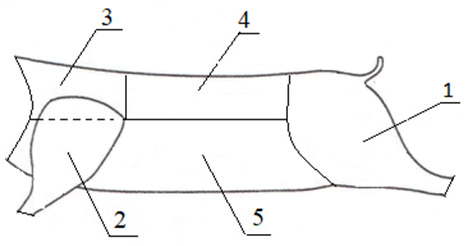
Primal cuts of the wild boar carcass: (1) ham, (2) shoulder, (3) neck, (4) loin and (5) spare ribs with belly.

Carcasses were split along the spinal column into two half-carcasses which were then divided into the following primal cuts:

1. ham – the pelvic segment of the half-carcass with the proximal segment of the hind limb, separated from the front half of the carcass between the two lowest lumbar vertebrae and along the perimysium of the quadriceps femoris muscle, separated from the top along the medial sacral crest, and separated from the bottom at the tarsal joint;
2. shoulder – the proximal forelimb from the carpal joint, separated from the thoracic wall in a semicircular cut along the anatomical shape of the shoulder between the muscles connecting the forelimb and the thoracic wall – scapular cartilage was retained;
3. neck – the shoulder blade, separated from the front half of the carcass along the head dissection line at the atlanto-occipital joint, separated from the back between the fourth and fifth thoracic vertebrae, and separated from the bottom and top along the carcass split line;
4. loin – the lumbar region of the spinal column, separated from the front half of the carcass between the fourth and fifth thoracic vertebrae, separated from the back between the two lowest lumbar vertebrae, separated from the top along the carcass split line, and separated from the bottom with a straight cut parallel to the spinal column, 3 cm away from the lower edge of the LTL muscle – tenderloin was retained;
5. spare ribs with belly – the rib region of the half-carcass with abdominal muscles, separated from the top half of the carcass along the loin cut line, separated from the front along the shoulder and neck cut line, separated from the back along the ham cut line, and separated from the bottom along the carcass split line (Fig. 2).

Primal cuts were dissected into lean meat, bones and fat. Meat was divided into three quality classes: class I – meat suitable for human consumption, including large and small cuts; class II – meat containing thick tendons, membranes and blood spots, intended for the production of animal feed; class III – discarded meat, mostly meat from the bullet wound area which is not suitable for consumption due to lead contamination.

Primal cuts and the dissected meat, bones and fat were weighed to the nearest 0.01 kg. The percentage of primal cuts was calculated relative to carcass weight, and the percentage of lean meat, bones and fat was calculated relative to the weight of primal cuts and the carcass. The percentage of meat from each quality class was expressed by the total weight of meat in the primal cuts and the carcass

### 2.3 Chemical analyses

The chemical composition of meat was determined from the LTL muscle obtained from each carcass (approx. sample weight 200 g). The moisture content was determined by drying meat samples to constant weight at a temperature of 105 ${}^{\circ }$C; protein content was determined using Kjeldahl's method with a conversion factor of 6.25; fat content was determined using Soxhlet extraction with petroleum ether as a solvent; ash content was determined by sample mineralisation at a temperature of 550–600 ${}^{\circ }$C (AOAC, 1995); and collagen content was determined by hydroxyproline quantification based on standard PN-ISO 3496 (Polish Committee for Standardization, 2000), with a conversion factor of 7.25. All chemical analyses were performed in triplicate.

### 2.4 Statistical analysis

**Figure 3:**
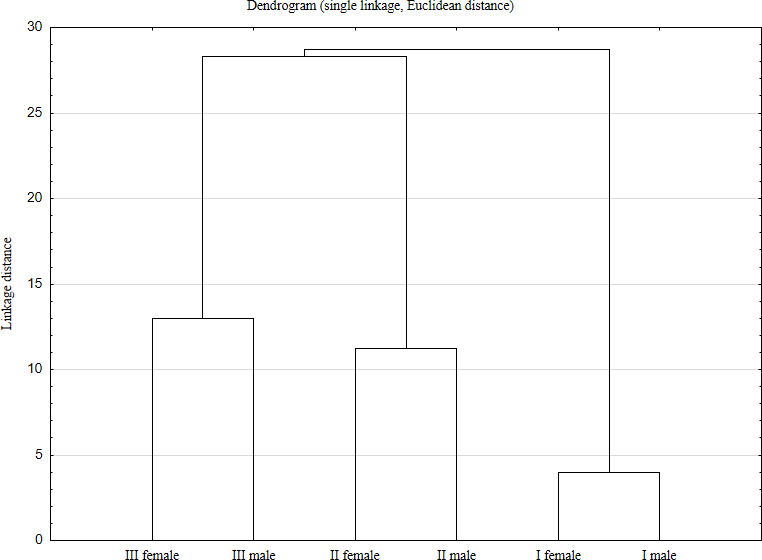
Complete-linkage dendrogram for female and male wild boars from different age groups (I piglets, <1 year; II yearlings, 1–2 years; III adults, 2–3 years).

Statistical analysis of the data was performed using Statistica 13.3 (TIBCO Software Inc., Palo Alto, CA., USA) software. The results are presented as arithmetic means and standard error of the mean (SE). The data were tested with respect to normal distribution (Shapiro–Wilk's test) and variance homogeneity (Levene's test). To examine the differences between the mean values obtained, an analysis of variance (ANOVA) was conducted as well as a Duncan's test. The significance level was set at 0.05. Mixed model ANOVA/ANCOVA (analysis of covariance; variation components module, with df (degrees of freedom) error calculated with the Satterthwaite method) was used to determine the effect of age (three levels: piglets, yearlings and adults) and sex (two levels: male and female). Age and sex were identified as fixed factors, and carcass weight was categorised as a random effect. Cluster analysis was used to classify objects into groups using carcass weight, weight and proportion of primal cuts; weight and proportion of meat, bones and fat in carcass; and chemical composition of the longissimus thoracis et lumborum muscle. This information is presented in the dendrogram in Fig. 3.

## 3 Results and discussion

### 3.1 Carcass and primal cuts' weights

Carcass weight varied significantly across the animals' age groups, whereas no significant differences were found between sexes within groups (Table 1). Similar carcass weights were reported for wild boars of a similar age (that were harvested in Poland) by Sales and Kotrba (2013) and Dzierżyńska-Cybulko and Fruziński (1997) in their review articles. On the other hand, the weights observed in the present study differ from those reported by Korzeniowski et al. (1991b): piglet carcasses were heavier (25 kg vs. 19 kg in Korzeniowski et al., 1991b, and this study respectively), whereas the carcasses of yearlings (37 kg vs. 40 kg in Korzeniowski et al., 1991b, and this study respectively) and older animals (55 kg vs. 62 kg in Korzeniowski et al., 1991b, and this study respectively) were lighter. Ludwiczak et al. (2020) also reported no effect of sex on piglet carcass weights; however, the carcasses of female boars were significantly lighter than males in the yearling group. The similar carcass weights might result from the similar body weights of male and female piglets, as reported by studies such as Borilova et al. (2016).

Generally, age affected carcass weights and all primal cuts' weights as well as the proportion of ham, neck and spare ribs with belly, whereas sex affected neck weight and the proportion of all primal cuts. There were no interactions between age and sex (Table 1). The weights of all primal cuts varied significantly across age groups, which resulted from the differences in carcass weight, and an increase in carcass weights and primal cuts' weights was observed along with animal age. No significant differences in the weights of cuts were found between sexes within groups, except for neck weight. An analysis of the percentage content of primal cuts in the carcass revealed more complex relationships. The largest primal cut (i.e. ham) accounted for more than 33 % (male) and 34 % (female) in group I, and its percentage was significantly higher than in the remaining groups. Significant differences between age groups were also noted in the neck for male boars and in the ribs with belly for female boars, where an increase in the proportion along with animal age was observed (Table 1). The age-related increase in the percentage content of the neck in the carcasses of wild boars is associated with digging up the ground in search for food, which contributes to neck muscle development (Korzeniowski et al., 1991b; Dzierżyńska-Cybulko and Fruziński, 1997). Korzeniowski et al. (1991b) also observed significant age-related differences in the weights of primal cuts in the carcass. With respect to the percentage content of primal cuts in the carcass, differences were only found in the neck between the oldest and the youngest animals. An increase in the percentage content of ham and shoulder meat in the carcass with increasing carcass weight was observed by Żochowska et al. (2004) in male boars; however, differences regarding other primal cuts were less pronounced or were absent. Significant sex-related differences were noted in the percentages of ham and shoulder meat in the carcasses of 2-year-old boars. It was also demonstrated that habitat conditions and food availability had a greater influence on the percentage content of primal cuts in the carcass than sex (Żochowska-Kujawska et al., 2010b).

An analysis of the percentage content of primal cuts in the carcass revealed that ham was the largest cut, followed by shoulder, loin, spare ribs with belly and neck (Table 1). Such proportions have also been documented in the literature. A comparison of the available data on the most valuable cuts in boar carcasses shows that the ham content reported by other authors is similar (Korzeniowski et al., 1991b; Żochowska et al., 2004; Żochowska-Kujawska et al., 2010b) or lower (Żmijewski and Korzeniowski, 2000; Żmijewski et al., 2007; Skewes et al., 2008) than that noted in our study. In all of the above-mentioned studies, the loin content of boar carcasses was lower than that determined in our study.

**Table 1:** Weight and percentage content (with respect to the carcass weight) of primal cuts in wild boar carcasses (mean ± SE).

Primal cuts	I (piglets)		II (yearlings)		III (adults)		P value		
	<1 year		1–2 years		2–3 years				
							Age (A)	Sex (S)	A×S
Carcass (kg)	19.04${}^{\mathrm{cx}}$ ± 1.39	18.40${}^{\mathrm{cx}}$ ± 1.45	39.95${}^{\mathrm{bx}}$ ± 2.19	40.82${}^{\mathrm{bx}}$ ± 1.31	62.68${}^{\mathrm{ax}}$ ± 2.86	62.21${}^{\mathrm{ax}}$ ± 3.85	${}^{***}$	NS	NS
Ham (kg)	6.30${}^{\mathrm{cx}}$ ± 0.15	6.30${}^{\mathrm{cx}}$ ± 0.27	12.05${}^{\mathrm{bx}}$ ± 0.41	12.90${}^{\mathrm{bx}}$ ± 0.33	18.70${}^{\mathrm{ax}}$ ± 0.70	19.33${}^{\mathrm{ax}}$ ± 1.10	${}^{***}$	NS	NS
Loin (kg)	3.46${}^{\mathrm{cx}}$ ± 0.17	3.59${}^{\mathrm{cx}}$ ± 0.25	7.41${}^{\mathrm{bx}}$ ± 0.33	7.58${}^{\mathrm{bx}}$ ± 0.20	11.57${}^{\mathrm{ax}}$ ± 0.69	12.90${}^{\mathrm{ax}}$ ± 0.80	${}^{***}$	NS	NS
Shoulder (kg)	3.78${}^{\mathrm{cx}}$ ± 0.29	3.46${}^{\mathrm{cx}}$ ± 0.35	8.13${}^{\mathrm{bx}}$ ± 0.37	7.64${}^{\mathrm{bx}}$ ± 0.45	12.32${}^{\mathrm{ax}}$ ± 0.78	11.01${}^{\mathrm{ax}}$ ± 0.82	${}^{***}$	NS	NS
Neck (kg)	2.21${}^{\mathrm{cx}}$ ± 0.15	1.95${}^{\mathrm{cx}}$ ± 0.09	5.83${}^{\mathrm{bx}}$ ± 0.28	4.99${}^{\mathrm{by}}$ ± 0.21	8.85${}^{\mathrm{ax}}$ ± 0.56	7.26${}^{\mathrm{ay}}$ ± 0.73	${}^{***}$	${}^{***}$	NS
Ribs${}^{f}$ (kg)	3.29${}^{\mathrm{cx}}$ ± 0.26	3.10${}^{\mathrm{cx}}$ ± 0.19	6.53${}^{\mathrm{bx}}$ ± 0.37	7.71${}^{\mathrm{bx}}$ ± 0.39	11.24${}^{\mathrm{ax}}$ ± 0.60	11.71${}^{\mathrm{ax}}$ ± 0.75	${}^{***}$	NS	NS
Ham (%)	33.09${}^{\mathrm{ay}}$	34.24${}^{\mathrm{ax}}$	30.16${}^{\mathrm{by}}$	31.60${}^{\mathrm{bx}}$	29.83${}^{\mathrm{by}}$	31.07${}^{\mathrm{bx}}$	${}^{***}$	${}^{***}$	NS
Loin (%)	18.17${}^{\mathrm{ax}}$	19.51${}^{\mathrm{ax}}$	18.55${}^{\mathrm{ax}}$	18.57${}^{\mathrm{ax}}$	18.46${}^{\mathrm{ay}}$	20.74${}^{\mathrm{ax}}$	NS	${}^{*}$	NS
Shoulder (%)	19.85${}^{\mathrm{ax}}$	18.80${}^{\mathrm{ay}}$	20.35${}^{\mathrm{ax}}$	18.72${}^{\mathrm{ay}}$	19.66${}^{\mathrm{ax}}$	17.70${}^{\mathrm{ay}}$	NS	${}^{***}$	NS
Neck (%)	11.61${}^{\mathrm{bx}}$	10.60${}^{\mathrm{bx}}$	14.59${}^{\mathrm{ax}}$	12.22${}^{\mathrm{ay}}$	14.12${}^{\mathrm{ay}}$	11.67${}^{\mathrm{abx}}$	${}^{***}$	${}^{***}$	NS
Ribs${}^{f}$ (%)	17.28${}^{\mathrm{abx}}$	16.85${}^{\mathrm{bx}}$	16.35${}^{\mathrm{by}}$	18.89${}^{\mathrm{ax}}$	17.93${}^{\mathrm{bx}}$	18.82${}^{\mathrm{ax}}$	${}^{*}$	${}^{*}$	NS

${}^{a,\;b,\;c}$ Different letters in rows indicate significant differences (P≤0.05) between values obtained for animals of the same sex at different ages. ${}^{x,\;y}$ Different letters in rows indicate significant differences (P≤0.05) between values obtained for animals of the same age with different sexes. “${}^{***}$” denotes P<0.001, “${}^{**}$” denotes P<0.01, “${}^{*}$” denotes P<0.05 and “NS” denotes no significant difference (P>0.05). ${}^{f}$ Spare ribs with belly. 

 denotes male. 

 denotes female.

### 3.2 Tissue composition of carcasses and primal cuts

Carcass value and processing suitability are largely determined by the content of lean meat, in particular class I meat intended for human consumption. On average, more than 12 kg of lean meat was obtained from piglet carcasses, meat yield was over twofold higher in yearlings, and it exceeded 40 kg in adults. Similar trends were observed in the weights of bones and fat (Table 2). The differences were statistically significant and resulted from age-related differences in carcass weight. The age of animals affected the weight of lean meat, fat and bones, whereas sex affected bone and fat weights. The fat weight was higher in the carcasses of females from the yearling and adult (II and III) groups compared with male carcasses. An analysis of the percentage content of lean meat in the carcasses revealed significant age-related and sex-related differences (Table 2). The carcasses of males from groups II and III had a significantly higher proportion of meat (above 70 %) compared with males from group I. In the groups of yearlings and adults, significant sex-related differences in the percentages of lean meat, bones and fat were noted: males had a higher proportion of lean meat and bones, whereas females had a higher proportion of fat. Similar results were reported by Aymerich et al. (2019) for swine: male carcasses were leaner than female carcasses. Moreover, the proportion of fat in female boars increased with age, and significant differences were noted between female piglets and older animals. Higher fat deposition in female wild boars might be associated with the role of fat tissue in the production of female hormones, which enable the animals to get pregnant, as well as serving as an energy source during pregnancy and the nursing period (Coelho et al., 2013; Eaton and Sethi, 2019).

**Table 2:** Tissue composition of wild boar carcasses in weight and percentage with respect to the carcass weight (mean ± SE).

Primal cuts	I (piglets)		II (yearlings)		III (adults)		P value		
	<1 year		1–2 years		2–3 years				
							Age (A)	Sex (S)	A×S
Lean meat (kg)	12.88${}^{\mathrm{cx}}$ ± 0.85	12.11${}^{\mathrm{cx}}$ ± 0.91	28.28${}^{\mathrm{bx}}$ ± 1.40	26.77${}^{\mathrm{bx}}$ ± 1.10	44.51${}^{\mathrm{ax}}$ ± 2.15	40.57${}^{\mathrm{ay}}$ ± 1.97	${}^{***}$	NS	NS
Bones (kg)	4.89${}^{\mathrm{cx}}$ ± 0.30	4.65${}^{\mathrm{cx}}$ ± 0.23	9.44${}^{\mathrm{bx}}$ ± 0.56	8.63${}^{\mathrm{bx}}$ ± 0.91	12.74${}^{\mathrm{ax}}$ ± 0.79	11.43${}^{\mathrm{ay}}$ ± 0.68	${}^{***}$	${}^{*}$	NS
Fat (kg)	1.23${}^{\mathrm{cx}}$ ± 0.11	1.64${}^{\mathrm{cx}}$ ± 0.09	2.21${}^{\mathrm{by}}$ ± 0.30	5.38${}^{\mathrm{bx}}$ ± 0.75	5.26${}^{\mathrm{ay}}$ ± 0.61	10.06${}^{\mathrm{ax}}$ ± 1.15	${}^{***}$	${}^{***}$	NS
Lean meat (%)	67.65${}^{\mathrm{bx}}$	65.82${}^{\mathrm{bx}}$	70.79${}^{\mathrm{ax}}$	65.58${}^{\mathrm{by}}$	71.01${}^{\mathrm{ax}}$	65.21${}^{\mathrm{by}}$	NS	${}^{***}$	NS
Bones (%)	25.68${}^{\mathrm{ax}}$	25.27${}^{\mathrm{ax}}$	23.63${}^{\mathrm{bx}}$	21.14${}^{\mathrm{by}}$	20.33${}^{\mathrm{cx}}$	18.37${}^{\mathrm{cy}}$	${}^{***}$	${}^{***}$	NS
Fat (%)	6.46${}^{\mathrm{bx}}$	8.91${}^{\mathrm{bx}}$	5.53${}^{\mathrm{by}}$	13.18${}^{\mathrm{ax}}$	8.39${}^{\mathrm{by}}$	16.17${}^{\mathrm{ax}}$	${}^{***}$	${}^{***}$	NS

${}^{a,\;b,\;c}$ Different letters in rows indicate significant differences (P≤0.05) between values obtained for animals of the same sex at different ages. ${}^{x,\;y}$ Different letters in rows indicate significant differences (P≤0.05) between values obtained for animals of the same age with different sexes. “${}^{***}$” denotes P<0.001, “${}^{**}$” denotes P<0.01, “${}^{*}$” denotes P<0.05 and “NS” denotes no significant difference (P>0.05). 

 denotes male. 

 denotes female.

**Table 3:** Tissue composition of primal cuts of boar carcasses in weight and percentage with respect to the cut weight (mean ± SE).

Primal cuts	I (piglets)		II (yearlings)		III (adults)		P value		
	<1 year		1–2 years		2–3 years				
							Age (A)	Sex (S)	A×S
Ham									
Lean meat (kg)	4.38${}^{\mathrm{cx}}$ ± 0.18	4.40${}^{\mathrm{cx}}$ ± 0.17	8.89${}^{\mathrm{bx}}$ ± 0.41	9.04${}^{\mathrm{bx}}$ ± 0.28	13.98${}^{\mathrm{ax}}$ ± 0.81	13.49${}^{\mathrm{ax}}$ ± 0.89	${}^{***}$	NS	NS
Bones (kg)	1.43${}^{\mathrm{cx}}$ ± 0.04	1.35${}^{\mathrm{cx}}$ ± 0.04	2.46${}^{\mathrm{bx}}$ ± 0.10	2.37${}^{\mathrm{bx}}$ ± 0.09	3.22${}^{\mathrm{ax}}$ ± 0.11	3.10${}^{\mathrm{ax}}$ ± 0.09	${}^{***}$	NS	NS
Fat (kg)	0.49${}^{\mathrm{bx}}$ ± 0.10	0.55${}^{\mathrm{cx}}$ ± 0.11	0.70${}^{\mathrm{by}}$ ± 0.13	1.49${}^{\mathrm{bx}}$ ± 0.32	1.50${}^{\mathrm{ay}}$ ± 0.35	2.74${}^{\mathrm{ax}}$ ± 0.60	${}^{***}$	${}^{**}$	NS
Lean meat (%)	69.52${}^{\mathrm{bx}}$	69.84${}^{\mathrm{ax}}$	73.78${}^{\mathrm{ax}}$	70.08${}^{\mathrm{ay}}$	74.76${}^{\mathrm{ax}}$	69.79${}^{\mathrm{ay}}$	${}^{*}$	${}^{**}$	NS
Bones (%)	22.70${}^{\mathrm{ax}}$	21.43${}^{\mathrm{ay}}$	20.41${}^{\mathrm{bx}}$	18.37${}^{\mathrm{by}}$	17.22${}^{\mathrm{cx}}$	16.04${}^{\mathrm{cy}}$	${}^{***}$	${}^{***}$	NS
Fat (%)	7.78${}^{\mathrm{ax}}$	8.73${}^{\mathrm{bx}}$	5.81${}^{\mathrm{ay}}$	11.55${}^{\mathrm{abx}}$	8.02${}^{\mathrm{ay}}$	14.17${}^{\mathrm{ax}}$	NS	${}^{***}$	NS
Loin									
Lean meat (kg)	2.14${}^{\mathrm{cx}}$ ± 0.13	2.05${}^{\mathrm{cx}}$ ± 0.10	4.74${}^{\mathrm{bx}}$ ± 0.25	4.42${}^{\mathrm{bx}}$ ± 0.21	7.01${}^{\mathrm{ax}}$ ± 0.40	6.99${}^{\mathrm{ax}}$ ± 0.49	${}^{***}$	NS	NS
Bones (kg)	1.02${}^{\mathrm{cx}}$ ± 0.05	1.15${}^{\mathrm{cx}}$ ± 0.04	2.00${}^{\mathrm{bx}}$ ± 0.10	1.86${}^{\mathrm{bx}}$ ± 0.08	2.89${}^{\mathrm{ax}}$ ± 0.13	2.64${}^{\mathrm{ax}}$ ± 0.11	${}^{***}$	NS	NS
Fat (kg)	0.29${}^{\mathrm{ax}}$ ± 0.06	0.39${}^{\mathrm{bx}}$ ± 0.09	0.66${}^{\mathrm{bx}}$ ± 0.15	1.28${}^{\mathrm{aby}}$ ± 0.26	1.63${}^{\mathrm{cy}}$ ± 0.39	3.26${}^{\mathrm{ax}}$ ± 0.41	${}^{***}$	${}^{***}$	NS
Lean meat (%)	61.85${}^{\mathrm{abx}}$	57.10${}^{\mathrm{aby}}$	63.97${}^{\mathrm{ax}}$	58.31${}^{\mathrm{ay}}$	60.59${}^{\mathrm{bx}}$	54.19${}^{\mathrm{by}}$	${}^{**}$	${}^{***}$	NS
Bones (%)	29.48${}^{\mathrm{ay}}$	32.03${}^{\mathrm{ax}}$	26.99${}^{\mathrm{bx}}$	24.54${}^{\mathrm{bx}}$	24.98${}^{\mathrm{bx}}$	20.47${}^{\mathrm{cy}}$	${}^{***}$	NS	NS
Fat (%)	8.38${}^{\mathrm{bx}}$	10.86${}^{\mathrm{cx}}$	8.91${}^{\mathrm{by}}$	16.89${}^{\mathrm{bx}}$	14.09${}^{\mathrm{ay}}$	25.27${}^{\mathrm{ax}}$	${}^{***}$	${}^{***}$	NS
Shoulder									
Lean meat (kg)	2.68${}^{\mathrm{cx}}$ ± 0.15	2.41${}^{\mathrm{cx}}$ ± 0.17	5.98${}^{\mathrm{bx}}$ ± 0.29	5.48${}^{\mathrm{bx}}$ ± 0.34	9.11${}^{\mathrm{ax}}$ ± 0.61	7.60${}^{\mathrm{ay}}$ ± 0.58	${}^{***}$	${}^{**}$	NS
Bones (kg)	0.84${}^{\mathrm{cx}}$ ± 0.05	0.75${}^{\mathrm{cx}}$ ± 0.05	1.75${}^{\mathrm{bx}}$ ± 0.11	1.47${}^{\mathrm{by}}$ ± 0.08	2.27${}^{\mathrm{ax}}$ ± 0.10	1.96${}^{\mathrm{ay}}$ ± 0.11	${}^{***}$	${}^{**}$	NS
Fat (kg)	0.26${}^{\mathrm{bx}}$ ± 0.02	0.30${}^{\mathrm{bx}}$ ± 0.03	0.40${}^{\mathrm{bx}}$ ± 0.07	0.68${}^{\mathrm{bx}}$ ± 0.11	0.90${}^{\mathrm{ay}}$ ± 0.09	1.38${}^{\mathrm{ax}}$ ± 0.17	${}^{***}$	${}^{*}$	NS
Lean meat (%)	70.90${}^{\mathrm{ax}}$	69.65${}^{\mathrm{ax}}$	73.55${}^{\mathrm{ax}}$	71.73${}^{\mathrm{ax}}$	73.94${}^{\mathrm{ax}}$	69.03${}^{\mathrm{ay}}$	NS	${}^{*}$	NS
Bones (%)	22.22${}^{\mathrm{ax}}$	21.68${}^{\mathrm{ax}}$	21.53${}^{\mathrm{ax}}$	19.24${}^{\mathrm{by}}$	18.43${}^{\mathrm{bx}}$	17.80${}^{\mathrm{bx}}$	${}^{***}$	${}^{**}$	NS
Fat (%)	6.88${}^{\mathrm{ax}}$	8.67${}^{\mathrm{ax}}$	4.92${}^{\mathrm{ax}}$	8.90${}^{\mathrm{ax}}$	7.31${}^{\mathrm{ay}}$	12.53${}^{\mathrm{bx}}$	${}^{*}$	${}^{**}$	NS
Neck									
Lean meat (kg)	1.53${}^{\mathrm{cx}}$ ± 0.13	1.30${}^{\mathrm{cx}}$ ± 0.09	4.10${}^{\mathrm{bx}}$ ± 0.19	3.44${}^{\mathrm{by}}$ ± 0.22	6.53${}^{\mathrm{ax}}$ ± 0.41	5.01${}^{\mathrm{ay}}$ ± 0.36	${}^{***}$	${}^{***}$	NS
Bones (kg)	0.63${}^{\mathrm{cx}}$ ± 0.12	0.50${}^{\mathrm{cx}}$ ± 0.07	1.44${}^{\mathrm{bx}}$ ± 0.09	1.08${}^{\mathrm{by}}$ ± 0.06	1.95${}^{\mathrm{ax}}$ ± 0.09	1.62${}^{\mathrm{ay}}$ ± 0.11	${}^{***}$	${}^{***}$	NS
Fat (kg)	0.03${}^{\mathrm{bx}}$ ± 0.02	0.15${}^{\mathrm{bx}}$ ± 0.01	0.28${}^{\mathrm{ax}}$ ± 0.02	0.47${}^{\mathrm{ax}}$ ± 0.04	0.35${}^{\mathrm{ax}}$ ± 0.07	0.61${}^{\mathrm{ay}}$ ± 0.03	${}^{***}$	${}^{**}$	NS
Lean meat (%)	69.23${}^{\mathrm{bx}}$	66.67${}^{\mathrm{ax}}$	70.33${}^{\mathrm{bx}}$	68.94${}^{\mathrm{ax}}$	73.79${}^{\mathrm{ax}}$	69.01${}^{\mathrm{ay}}$	${}^{*}$	${}^{**}$	NS
Bones (%)	28.51${}^{\mathrm{ax}}$	25.64${}^{\mathrm{ay}}$	24.70${}^{\mathrm{bx}}$	21.64${}^{\mathrm{by}}$	22.03${}^{\mathrm{cx}}$	22.31${}^{\mathrm{bx}}$	${}^{***}$	${}^{**}$	NS
Fat (%)	1.36${}^{\mathrm{by}}$	7.69${}^{\mathrm{ax}}$	4.80${}^{\mathrm{ay}}$	9.42${}^{\mathrm{ax}}$	3.95${}^{\mathrm{aby}}$	8.40${}^{\mathrm{ax}}$	${}^{*}$	${}^{***}$	NS
Spare ribs with belly									
Lean meat (kg)	2.15${}^{\mathrm{ax}}$ ± 0.13	1.95${}^{\mathrm{ax}}$ ± 0.09	4.57${}^{\mathrm{bx}}$ ± 0.21	4.39${}^{\mathrm{bx}}$ ± 0.25	7.88${}^{\mathrm{cx}}$ ± 0.40	7.48${}^{\mathrm{cx}}$ ± 0.37	${}^{***}$	NS	NS
Bones (kg)	0.97${}^{\mathrm{cx}}$ ± 0.05	0.90${}^{\mathrm{bx}}$ ± 0.07	1.79${}^{\mathrm{bx}}$ ± 0.04	1.85${}^{\mathrm{ax}}$ ± 0.05	2.41${}^{\mathrm{ax}}$ ± 0.11	2.11${}^{\mathrm{ay}}$ ± 0.09	${}^{***}$	NS	NS
Fat (kg)	0.16${}^{\mathrm{bx}}$ ± 0.06	0.25${}^{\mathrm{cx}}$ ± 0.39	0.17${}^{\mathrm{by}}$ ± 0.06	1.46${}^{\mathrm{bx}}$ ± 0.70	0.88${}^{\mathrm{ay}}$ ± 0.45	2.07${}^{\mathrm{ax}}$ ± 1.03	${}^{***}$	${}^{***}$	NS
Lean meat (%)	65.35${}^{\mathrm{ax}}$	62.90${}^{\mathrm{ax}}$	69.98${}^{\mathrm{ax}}$	56.94${}^{\mathrm{by}}$	70.11${}^{\mathrm{ax}}$	63.88${}^{\mathrm{ay}}$	NS	${}^{***}$	NS
Bones (%)	29.48${}^{\mathrm{ax}}$	29.03${}^{\mathrm{ax}}$	27.41${}^{\mathrm{ax}}$	23.99${}^{\mathrm{by}}$	21.44${}^{\mathrm{bx}}$	18.02${}^{\mathrm{cy}}$	${}^{***}$	${}^{*}$	NS
Fat (%)	4.86${}^{\mathrm{abx}}$	8.06${}^{\mathrm{bx}}$	2.60${}^{\mathrm{by}}$	18.94${}^{\mathrm{ax}}$	7.83${}^{\mathrm{ay}}$	17.68${}^{\mathrm{ax}}$	${}^{**}$	${}^{***}$	NS

${}^{a,\;b,\;c}$ Different letters in rows indicate significant differences (P≤0.05) between values obtained for animals of the same sex at different ages. ${}^{x,\;y}$ Different letters in rows indicate significant differences (P≤0.05) between values obtained for animals of the same age with different sexes. “${}^{***}$” denotes P<0.001, “${}^{**}$” denotes P<0.01, “${}^{*}$” denotes P<0.05 and “NS” denotes no significant difference (P>0.05). 

 denotes male. 

 denotes female.

An analysis of the tissue composition of boar carcasses across age groups indicated that the proportion of bones decreases with animal age regardless of sex (Table 2), due to the increasing muscle and fat weight and proportion in carcasses. Significant differences in the percentage of lean meat and bones in boar carcasses were also reported by Korzeniowski et al. (1991b), where the meat content increased and the bone content decreased with increasing carcass weight. In the present study, the percentage content of lean meat and fat was higher, whereas the percentage of bones was lower than the values reported by other authors (Korzeniowski et al., 1991b; Żmijewski and Korzeniowski, 2000).

Both age and sex affected the tissue composition (weight and proportion) of primal cuts (Table 3). It was noted that the percentage content of lean meat in all cuts was the highest in the carcasses of males from groups II and III, and the percentage of fat was the highest in the carcasses of females from groups II and III. An analysis of the tissue composition of primal cuts across the age groups of boars revealed significant differences between group I and group II and between group I and group III; in most cases, the fat content was lower and bone content was higher in the primal cuts of boar carcasses from group I. In all groups, the meat content was highest in the ham (from 69.5 % to 74.8 %) and shoulder cuts (from 69.0 % to 73.9 %), and lowest in the loin cut (from 54.2 % to 61.9 %) (Table 3). Similar trends were observed by Korzeniowski et al. (1991b) and Żmijewski and Korzeniowski (2000). However, the above-mentioned authors noted a higher meat and bone content and a considerably lower fat content in the analysed primal cuts. Lower bone content and high meat yield were reported by Ristić et al. (1987). In a study by Żmijewski et al. (2007), where the average carcass weight reached 43.8 kg and was comparable to the carcass weight determined in group II in the present study, most primal cuts had a higher lean meat content, similar bone content and lower fat content. The fat proportion increased with animal age in primal cuts, and it was higher in females than in males starting from the yearling group (Table 3). Sex-related differences in the proportion of fat in primal cuts were also noted by Aymerich et al. (2019) in swine, where ham from male pigs was leaner than that from female pigs.

**Table 4:** Characteristics of wild boar meat in weight and percentage of lean meat (mean ± SE).

Primal cuts	I (piglets)		II (yearlings)		III (adults)		P value		
	<1 year		1–2 years		2–3 years				
							Age (A)	Sex (S)	A×S
Lean meat (kg)	12.88${}^{\mathrm{cx}}$ ± 0.85	12.11${}^{\mathrm{cx}}$ ± 0.91	28.28${}^{\mathrm{bx}}$ ± 1.40	26.77${}^{\mathrm{bx}}$ ± 1.10	44.51${}^{\mathrm{ax}}$ ± 2.15	40.57${}^{\mathrm{ay}}$ ± 1.97	${}^{***}$	NS	NS
Class I (kg)	10.90${}^{\mathrm{cx}}$ ± 1.13	10.15${}^{\mathrm{cx}}$ ± 1.10	23.49${}^{\mathrm{bx}}$ ± 1.95	22.06${}^{\mathrm{bx}}$ ± 2.05	37.47${}^{\mathrm{ax}}$ ± 2.90	35.57${}^{\mathrm{ax}}$ ± 3.39	${}^{***}$	NS	NS
Class II (kg)	0.81${}^{\mathrm{bx}}$ ± 0.21	0.40${}^{\mathrm{bx}}$ ± 0.26	2.64${}^{\mathrm{ax}}$ ± 0.31	2.50${}^{\mathrm{ax}}$ ± 0.40	2.81${}^{\mathrm{ax}}$ ± 0.62	2.06${}^{\mathrm{ax}}$ ± 0.89	${}^{***}$	NS	NS
Class III (kg)	1.14${}^{\mathrm{ax}}$ ± 0.36	1.40${}^{\mathrm{ax}}$ ± 0.60	2.10${}^{\mathrm{ax}}$ ± 0.85	2.16${}^{\mathrm{ax}}$ ± 0.94	4.18${}^{\mathrm{ax}}$ ± 2.11	2.87${}^{\mathrm{ax}}$ ± 1.54	NS	NS	NS
Class I (%)	84.63${}^{\mathrm{ax}}$	83.82${}^{\mathrm{ax}}$	83.06${}^{\mathrm{ax}}$	82.41${}^{\mathrm{ax}}$	84.18${}^{\mathrm{ax}}$	87.68${}^{\mathrm{ax}}$	NS	NS	NS
Class II (%)	6.29${}^{\mathrm{bx}}$	3.30${}^{\mathrm{bx}}$	9.34${}^{\mathrm{ax}}$	9.34${}^{\mathrm{ax}}$	6.31${}^{\mathrm{bx}}$	5.08${}^{\mathrm{bx}}$	${}^{*}$	NS	NS
Class III (%)	8.85${}^{\mathrm{ax}}$	11.56${}^{\mathrm{ax}}$	7.43${}^{\mathrm{ax}}$	8.07${}^{\mathrm{ax}}$	9.39${}^{\mathrm{ax}}$	7.07${}^{\mathrm{ax}}$	NS	NS	NS

${}^{a,\;b,\;c}$ Different letters in rows indicate significant differences (P≤0.05) between values obtained for animals of the same sex at different ages. ${}^{x,\;y}$ Different letters in rows indicate significant differences (P≤0.05) between values obtained for animals of the same age with different sexes. “${}^{***}$” denotes P<0.001, “${}^{**}$” denotes P<0.01, “${}^{*}$” denotes P<0.05 and “NS” denotes no significant difference (P>0.05). 

 denotes male. 

 denotes female.

Certain general trends in the percentage content of primal cuts in boar carcasses can be observed when analysing data from the literature, but a direct comparison of research findings is not possible due to considerable differences in the age, body weight, sex, habitat and health status of the wild boars analysed. Differences in the division of a carcass into primal cuts and cutting lines (i.e. including or excluding the head, front of the neck and subcutaneous fat) are other important considerations. All of the above factors affect the proportions of primal cuts in the carcass, as confirmed by Żochowska-Kujawska et al. (2010b).

**Table 5:** Characteristics of wild boar meat from different primal cuts in weight and percentage of lean meat (mean ± SE).

Primal cuts	I (piglets)		II (yearlings)		III (adults)		P value		
	<1 year		1–2 years		2–3 years				
							Age (A)	Sex (S)	A×S
Ham									
Class I (kg)	4.19${}^{\mathrm{cx}}$ ± 0.19	4.10${}^{\mathrm{cx}}$ ± 0.17	7.82${}^{\mathrm{bx}}$ ± 0.24	8.25${}^{\mathrm{bx}}$ ± 0.35	12.51${}^{\mathrm{ax}}$ ± 0.58	12.39${}^{\mathrm{ax}}$ ± 0.49	${}^{***}$	NS	NS
Class II (kg)	0.15${}^{\mathrm{bx}}$ ± 0.01	0.30${}^{\mathrm{ax}}$ ± 0.15	0.52${}^{\mathrm{ax}}$ ± 0.03	0.53${}^{\mathrm{ax}}$ ± 0.03	0.59${}^{\mathrm{ax}}$ ± 0.02	0.60${}^{\mathrm{ax}}$ ± 0.03	${}^{**}$	NS	NS
Class III (kg)	0.03${}^{\mathrm{bx}}$ ± 0.01	0.00${}^{\mathrm{bx}}$ ± 0.00	0.55${}^{\mathrm{ax}}$ ± 0.21	0.25${}^{\mathrm{aby}}$ ± 0.17	0.86${}^{\mathrm{ax}}$ ± 0.42	0.48${}^{\mathrm{ay}}$ ± 0.36	${}^{***}$	${}^{*}$	NS
Class I (%)	95.66${}^{\mathrm{ax}}$	93.18${}^{\mathrm{ax}}$	87.96${}^{\mathrm{ax}}$	91.26${}^{\mathrm{ax}}$	89.48${}^{\mathrm{ax}}$	91.85${}^{\mathrm{ax}}$	NS	NS	NS
Class II (%)	3.42${}^{\mathrm{by}}$	6.82${}^{\mathrm{ax}}$	5.85${}^{\mathrm{ax}}$	5.86${}^{\mathrm{abx}}$	4.22${}^{\mathrm{abx}}$	4.45${}^{\mathrm{bx}}$	NS	NS	NS
Class III (%)	0.68${}^{\mathrm{bx}}$	0.00${}^{\mathrm{bx}}$	6.19${}^{\mathrm{ax}}$	2.77${}^{\mathrm{aby}}$	6.15${}^{\mathrm{bx}}$	3.56${}^{\mathrm{ay}}$	${}^{***}$	${}^{*}$	NS
Loin									
Class I (kg)	1.92${}^{\mathrm{ax}}$ ± 0.17	1.90${}^{\mathrm{ax}}$ ± 0.14	3.97${}^{\mathrm{bx}}$ ± 0.25	3.84${}^{\mathrm{bx}}$ ± 0.27	6.14${}^{\mathrm{cx}}$ ± 0.33	6.58${}^{\mathrm{cx}}$ ± 0.29	${}^{***}$	NS	NS
Class II (kg)	0.20${}^{\mathrm{ax}}$ ± 0.08	0.15${}^{\mathrm{ax}}$ ± 0.09	0.65${}^{\mathrm{bx}}$ ± 0.13	0.57${}^{\mathrm{bx}}$ ± 0.10	0.87${}^{\mathrm{bx}}$ ± 0.17	0.41${}^{\mathrm{by}}$ ± 0.09	${}^{***}$	${}^{***}$	NS
Class III (kg)	0.02${}^{\mathrm{ax}}$ ± 0.01	0.00${}^{\mathrm{ay}}$ ± 0.00	0.12${}^{\mathrm{bx}}$ ± 0.01	0.00${}^{\mathrm{ay}}$ ± 0.00	0.00${}^{\mathrm{ax}}$ ± 0.00	0.00${}^{\mathrm{ax}}$ ± 0.00	${}^{***}$	${}^{***}$	NS
Class I (%)	89.72${}^{\mathrm{ax}}$	92.68${}^{\mathrm{ax}}$	83.76${}^{\mathrm{ax}}$	86.88${}^{\mathrm{ax}}$	87.59${}^{\mathrm{ax}}$	94.13${}^{\mathrm{ax}}$	NS	NS	NS
Class II (%)	9.35${}^{\mathrm{ax}}$	7.32${}^{\mathrm{ax}}$	13.71${}^{\mathrm{bx}}$	12.90${}^{\mathrm{bx}}$	12.41${}^{\mathrm{bx}}$	5.87${}^{\mathrm{ay}}$	${}^{***}$	${}^{***}$	NS
Class III (%)	0.93${}^{\mathrm{bx}}$	0.00${}^{\mathrm{ay}}$	2.53${}^{\mathrm{ax}}$	0.00${}^{\mathrm{ay}}$	0.00${}^{\mathrm{cx}}$	0.00${}^{\mathrm{ax}}$	${}^{***}$	${}^{***}$	NS
Shoulder									
Class I (kg)	2.38${}^{\mathrm{cx}}$ ± 0.16	2.20${}^{\mathrm{cx}}$ ± 0.10	5.22${}^{\mathrm{bx}}$ ± 0.31	4.60${}^{\mathrm{bx}}$ ± 0.37	8.50${}^{\mathrm{ax}}$ ± 0.46	6.94${}^{\mathrm{ay}}$ ± 0.32	${}^{***}$	${}^{**}$	NS
Class II (kg)	0.30${}^{\mathrm{ax}}$ ± 0.01	0.20${}^{\mathrm{bx}}$ ± 0.01	0.67${}^{\mathrm{ax}}$ ± 0.21	0.87${}^{\mathrm{ax}}$ ± 0.25	0.60${}^{\mathrm{ax}}$ ± 0.15	0.65${}^{\mathrm{abx}}$ ± 0.10	${}^{**}$	NS	NS
Class III (kg)	0.00${}^{\mathrm{bx}}$ ± 0.00	0.00${}^{\mathrm{ax}}$ ± 0.00	0.08${}^{\mathrm{ax}}$ ± 0.04	0.00${}^{\mathrm{ay}}$ ± 0.00	0.00${}^{\mathrm{bx}}$ ± 0.00	0.00${}^{\mathrm{ax}}$ ± 0.00	${}^{**}$	${}^{*}$	NS
Class I (%)	88.81${}^{\mathrm{ax}}$	91.29${}^{\mathrm{ax}}$	87.29${}^{\mathrm{ax}}$	83.94${}^{\mathrm{ax}}$	93.30${}^{\mathrm{ax}}$	91.32${}^{\mathrm{ax}}$	NS	NS	NS
Class II (%)	11.19${}^{\mathrm{ax}}$	8.30${}^{\mathrm{bx}}$	11.20${}^{\mathrm{ax}}$	15.88${}^{\mathrm{ax}}$	6.59${}^{\mathrm{bx}}$	8.55${}^{\mathrm{bx}}$	${}^{*}$	NS	NS
Class III (%)	0.00${}^{\mathrm{bx}}$	0.00${}^{\mathrm{ax}}$	1.34${}^{\mathrm{ax}}$	0.00${}^{\mathrm{ay}}$	0.00${}^{\mathrm{bx}}$	0.00${}^{\mathrm{ax}}$	${}^{**}$	${}^{*}$	NS
Neck									
Class I (kg)	0.95${}^{\mathrm{bx}}$ ± 0.53	0.65${}^{\mathrm{bx}}$ ± 0.31	2.79${}^{\mathrm{ax}}$ ± 0.25	2.38${}^{\mathrm{ax}}$ ± 0.41	4.33${}^{\mathrm{ax}}$ ± 0.91	3.58${}^{\mathrm{ax}}$ ± 0.80	${}^{***}$	NS	NS
Class II (kg)	0.00${}^{\mathrm{bx}}$ ± 0.00	0.00${}^{\mathrm{ax}}$ ± 0.00	0.24${}^{\mathrm{ax}}$ ± 0.08	0.00${}^{\mathrm{ay}}$ ± 0.00	0.00${}^{\mathrm{bx}}$ ± 0.00	0.00${}^{\mathrm{ax}}$ ± 0.00	${}^{***}$	${}^{**}$	NS
Class III (kg)	0.58${}^{\mathrm{ax}}$ ± 0.36	0.65${}^{\mathrm{ax}}$ ± 0.25	1.04${}^{\mathrm{ax}}$ ± 0.51	1.05${}^{\mathrm{ax}}$ ± 0.39	2.19${}^{\mathrm{ax}}$ ± 1.06	1.42${}^{\mathrm{ax}}$ ± 0.73	NS	NS	NS
Class I (%)	62.09${}^{\mathrm{ax}}$	50.00${}^{\mathrm{ax}}$	68.05${}^{\mathrm{ax}}$	69.19${}^{\mathrm{ax}}$	66.31${}^{\mathrm{ax}}$	71.46${}^{\mathrm{ax}}$	NS	NS	NS
Class II (%)	0.00${}^{\;\mathrm{bx}}$	0.00${}^{\;\mathrm{ax}}$	5.85${}^{\mathrm{ax}}$	0.00${}^{\mathrm{ay}}$	0.00${}^{\;\mathrm{bx}}$	0.00${}^{\mathrm{ax}}$	${}^{***}$	${}^{**}$	NS
Class III (%)	37.91${}^{\mathrm{ax}}$	50.00${}^{\mathrm{ax}}$	25.37${}^{\mathrm{ax}}$	30.52${}^{\mathrm{ax}}$	33.54${}^{\mathrm{ax}}$	28.34${}^{\mathrm{ax}}$	NS	NS	NS
Spare ribs with belly									
Class I (kg)	1.46${}^{\mathrm{cx}}$ ± 0.08	1.30${}^{\mathrm{cx}}$ ± 0.06	3.69${}^{\mathrm{bx}}$ ± 0.11	2.99${}^{\mathrm{bx}}$ ± 0.10	5.99${}^{\mathrm{ax}}$ ± 0.20	6.08${}^{\mathrm{ax}}$ ± 0.28	${}^{***}$	NS	NS
Class II (kg)	0.16${}^{\mathrm{cx}}$ ± 0.02	0.20${}^{\mathrm{cx}}$ ± 0.01	0.56${}^{\mathrm{bx}}$ ± 0.01	0.53${}^{\mathrm{bx}}$ ± 0.02	0.75${}^{\mathrm{ax}}$ ± 0.15	0.40${}^{\mathrm{ay}}$ ± 0.17	${}^{***}$	${}^{*}$	NS
Class III (kg)	0.51${}^{\mathrm{bx}}$ ± 0.10	0.45${}^{\mathrm{bx}}$ ± 0.21	0.31${}^{\mathrm{by}}$ ± 0.19	0.86${}^{\mathrm{ax}}$ ± 0.21	1.13${}^{\mathrm{ax}}$ ± 0.30	0.97${}^{\mathrm{ax}}$ ± 0.13	${}^{***}$	NS	NS
Class I (%)	67.91${}^{\mathrm{cx}}$	66.67${}^{\mathrm{bx}}$	80.74${}^{\mathrm{bx}}$	68.11${}^{\mathrm{by}}$	76.02${}^{\mathrm{ay}}$	81.28${}^{\mathrm{ax}}$	${}^{***}$	${}^{**}$	NS
Class II (%)	7.44${}^{\mathrm{cy}}$	10.25${}^{\mathrm{bx}}$	12.25${}^{\mathrm{bx}}$	12.07${}^{\mathrm{ax}}$	9.52${}^{\mathrm{ax}}$	5.35${}^{\mathrm{cy}}$	${}^{***}$	NS	NS
Class III (%)	23.72${}^{\mathrm{ax}}$	23.08${}^{\mathrm{ax}}$	6.78${}^{\mathrm{by}}$	19.59${}^{\mathrm{bx}}$	14.34${}^{\mathrm{cx}}$	12.97${}^{\mathrm{cx}}$	${}^{***}$	${}^{**}$	NS

${}^{a,\;b,\;c}$ Different letters in rows indicate significant differences (P≤0.05) between values obtained for animals of the same sex at different ages. ${}^{x,\;y}$ Different letters in rows indicate significant differences (P≤0.05) between values obtained for animals of the same age with different sexes. “${}^{***}$” denotes P<0.001, “${}^{**}$” denotes P<0.01, “${}^{*}$” denotes P<0.05 and “NS” denotes no significant difference (P>0.05). 

 denotes male. 

 denotes female.

The weight of cuts suitable for human consumption (class I) is the main determinant of carcass value and processing suitability. The weight of class I cuts ranged from 10.2 to 37.5 kg and increased significantly with animal age (Table 4). The percentage of class I cuts in the total weight of meat in wild boar carcasses was relatively high and ranged from 82.4 % to 87.7 %. Moreover, the yield of class I meat was not significantly affected by carcass weight nor animal age (Table 4). The remaining cuts that are not suitable for human consumption are used in the production of animal feed (class II) or are discarded (class III). The total content of class II and class III meat in the boar carcass is 10 % to 20 % respectively, and their proportions vary widely, as demonstrated by high SE values (Table 4). The above variations can be attributed to differences in hunting conditions and hunting skill. The above factors also influence the extent of damage to carcass cuts. The highest percentage of class I meat (suitable for human consumption) was found in the most valuable cuts, including ham, loin and shoulder (from 84 % to 96 %), and the observed differences were not related to sex or age (Table 5). The highest percentage of class III meat was noted in the neck (from 25.4 % to 50.0 %) and spare ribs with belly (from 6.8 % to 23.7 %) cuts (Table 5). In an earlier study by Żmijewski et al. (2007), the highest percentage of meat of the lowest quality was obtained from the neck (32.0 %), spare ribs (16.8 %) and shoulder (16.2 %) areas. However, a different system of dividing cuts into quality classes were used; therefore, those results cannot be directly compared to the findings of the present study. The above cuts are at high risk of bullet damage because proper shot placement should prevent damage to primal cuts and ensure rapid bleeding and quick death (Janiszewski and Daszkiewicz, 2010; Tropiło and Kiszczak, 2008).

### 3.3 Chemical composition of the loin

The chemical composition of the loin (LTL muscle) is shown in Table 6. The age of animals significantly affected the protein and collagen content, whereas sex had no influence on the chemical composition of the muscle. These results partially resemble those presented by Ludwiczak et al. (2020), who showed that age and sex (in groups of wild boar piglets and yearlings) did not affect moisture and protein contents in the semimembranosus muscle and offal, but fat content was affected by both age and sex, and was greater in the meat and offal of yearlings and females. Postolache et al. (2010) and Dannenberger et al. (2013) found no sex-related differences in the proximate chemical composition of boar meat, which supports the findings of this study. Marsico et al. (2007), who analysed the proximate chemical composition of the LTL muscle in wild boars, reported a higher content of protein (25.9 %) and ash (1.2 %) and a lower content of moisture and fat compared with the values noted in this study. Similar protein and lower fat contents were reported by Strazdina et al. (2013) and Pedrazzoli et al. (2017), whereas Strazdina et al. (2015) reported lower protein and similar fat contents. In the 2- to 3-year-old boars examined by Postolache et al. (2010), the LTL muscle had a lower protein content in both sexes, a lower fat content in females and similar fat content in males compared with this study. In other studies, the content of protein and fat in the LTL muscle of boars with similar body weights was lower than in this study (Żmijewski and Korzeniowski, 2001; Szmańko et al., 2007; Razmaite et al., 2012; Amici et al., 2015; Kasprzyk, 2015). On the other hand, Duma-Kocan et al. (2019), reported a similar fat content (3.09 %) for yearlings to that noted in this study.

**Table 6:** Chemical composition of wild boar loin (longissimus thoracis et lumborum) muscle (mean ± SE).

Component	I (piglets)		II (yearlings)		III (adults)		P value		
	<1 year		1–2 years		2–3 years				
							Age (A)	Sex (S)	A×S
Moisture (%)	73.03${}^{\mathrm{ax}}$ ± 0.58	73.52${}^{\mathrm{ax}}$ ± 0.98	71.96${}^{\mathrm{ax}}$ ± 0.55	71.61${}^{\mathrm{ax}}$ ± 0.81	71.36${}^{\mathrm{ax}}$ ± 0.59	71.48${}^{\mathrm{ax}}$ ± 1.06	NS	NS	NS
Protein (%)	22.32${}^{\mathrm{bx}}$ ± 0.77	22.21${}^{\mathrm{bx}}$ ± 0.64	23.71${}^{\mathrm{abx}}$ ± 0.42	23.23${}^{\mathrm{abx}}$ ± 0.58	24.46${}^{\mathrm{ax}}$ ± 0.54	24.33${}^{\mathrm{ax}}$ ± 0.61	${}^{*}$	NS	NS
Fat (%)	3.62${}^{\mathrm{ax}}$ ± 0.80	3.22${}^{\mathrm{ax}}$ ± 0.62	3.33${}^{\mathrm{ax}}$ ± 0.44	4.05${}^{\mathrm{ax}}$ ± 0.54	3.03${}^{\mathrm{ax}}$ ± 0.48	3.04${}^{\mathrm{ax}}$ ± 0.21	NS	NS	NS
Ash (%)	1.03${}^{\mathrm{ax}}$ ± 0.06	1.03${}^{\mathrm{ax}}$ ± 0.07	1.00${}^{\mathrm{ax}}$ ± 0.15	1.06${}^{\mathrm{ax}}$ ± 0.13	1.14${}^{\mathrm{ax}}$ ± 0.03	1.13${}^{\mathrm{ax}}$ ± 0.06	NS	NS	NS
Collagen (%)	0.83${}^{\mathrm{ax}}$ ± 0.04	0.78${}^{\mathrm{ax}}$ ± 0.03	0.63${}^{\mathrm{bx}}$ ± 0.03	0.65${}^{\mathrm{bx}}$ ± 0.03	0.63${}^{\mathrm{bx}}$ ± 0.04	0.61${}^{\mathrm{bx}}$ ± 0.02	${}^{***}$	NS	NS

${}^{a,\;b,\;c}$ Different letters in rows indicate significant differences (P≤0.05) between values obtained for animals of the same sex at different ages. ${}^{x,\;y}$ Different letters in rows indicate significant differences (P≤0.05) between values obtained for animals of the same age with different sexes. “${}^{***}$” denotes P<0.001, “${}^{*}$” denotes P<0.05 and “NS” denotes no significant difference (P>0.05). 

 denotes male. 

 denotes female.

The LTL muscle of wild boars had a protein content ranging from 22 % to 24 %, and significantly higher values were noted for adult boars (group III) compared with piglets (group I). This increase in the protein content in boar meat with increasing carcass weight has also been reported by Kasprzyk et al. (2013) and Batorska et al. (2018).

Generally, game meat is considered to be a dietetic food, due to a fat content below 3 % (Batorska et al., 2018; Kasprzyk, 2015). However, in this study a relatively high fat content, ranging from 3.0 % to 4.0 %, was noted, and these values were higher than those reported by Żmijewski and Korzeniowski (2001), Marsico et al. (2007), Razmaite et al. (2012), Strazdina et al. (2013), Amici et al. (2015), and Pedrazzoli et al. (2017). This high fat content in the LTL muscle could result from the availability of high-quality food resources (maize, potatoes, cereal grain), as the boars foraged in arable fields. Fat weights and percentages were also relatively high in the loin (Table 3) and in the whole carcass (Table 2).

Differences were also observed in the collagen content, which was significantly higher in the LTL muscle of younger boars (0.8 %) compared with older animals (0.6 % in groups II and III). There were no significant sex-related differences in collagen content (Table 6), which does not confirm the opinion that meat from male boars has a higher connective tissue content (Dzierżyńska-Cybulko and Fruziński, 1997). The collagen content reported by other authors varies widely. In some studies, the collagen content of the LTL muscle was lower than in our study, 0.5 % (Oshima et al., 2009) and 0.4 % (Rede et al., 1986), whereas other authors reported a considerably higher collagen content, up to 1.8 % (Korzeniowski et al., 1991a; Dzierżyńska-Cybulko and Fruziński, 1997). Collagen (its concentration and solubility) might affect the quality of meat (Chriki et al., 2013; Dubost et al., 2013). The solubility of collagen decreases with animal age, due to increased cross-linking between collagen molecules, which might increase shear force values in muscles (Tatum, 2011; Modzelewska-Kapituła et al., 2014). Moreover, it has been noted that a lower collagen content in a muscle referred to its lower solubility, which, in turn, resulted in less tender meat (Modzelewska-Kapituła and Nogalski, 2014). However, to determine the influence of the differences in collagen concentration observed in this study between age groups, further analyses should be conducted, such as the determination of collagen solubility, shear force measurements and sensory analysis.

To show similarities between age and sex groups of wild boars a cluster analysis using carcass weight, weight and proportion of primal cuts; weight and proportion of meat, bones and fat in carcass; and chemical composition of LTL muscle was conducted, and the results are presented on Fig. 3. The complete-linkage dendrogram clearly shows that the age of the animals is the key factor that differentiates the carcass and meat value.

## 4 Conclusions

The results of this study indicate that carcass quality parameters, which determine its processing suitability, vary depending on the age and sex of wild boars and that the chemical composition of the LTL muscle is affected by animal age. The weights of carcass, primal cuts and meat increase with age; thus, harvesting older animals is more efficient and economically justified. Moreover, the carcasses of yearlings and adults are characterised by the highest processing suitability, which can be mainly attributed to the highest percentage of lean meat in the carcass and a moderate content of fat and bones. Female carcasses from the yearling and adult groups had lower values than male carcasses due to a higher content of fat in the carcasses and primal cuts. The chemical composition of the wild boar LTL muscle is most desirable in adult wild boars due to higher protein and lower collagen contents than in piglets, and it is unaffected by the animals' sex. However, in practice, the hunting regime is determined by the regulation of the population structure, and this is focused on preventing excessive damage to forests or agriculture not on wild boar meat quality.

## Data Availability

The data from this study can be obtained from the authors upon a reasonable request.

## References

[bib1.bib1] Amici A, Serrani F, Rossi CM, Primi R (2012). Increase in crop damage caused by wild boar (Sus scrofa L.): the “refuge effect”. Agronomy for Sustainable Development, Springer Verlag/EDP Sciences/INRA.

[bib1.bib2] Amici A, Cifuni GF, Conto M, Esposito L, Failla S (2015). Hunting area affects chemical and physical characteristics and fatty acid composition of wild boar (Sus scrofa) meat. Rend Fis Acc Lincei.

[bib1.bib3] AOAC (1995). Official methods of analysis of association of official analytical chemists.

[bib1.bib4] Aymerich P, Gasa J, Bonet J, Coma J, Solà-Oriol D (2019). The effects of sire line, sex, weight and marketing day on carcass fatness of non-castrated pigs. Livestock Sci.

[bib1.bib5] Batorska M, Więcek J, Kunowska-Slósarz M, Puppel K, Slósarz J, Gołębiewski M, Kuczyńska B, Popczyk B, Rekiel A, Balcerak M (2018). The effect of carcass weight on chemical characteristics and fatty acid composition of Longissimus dorsi and Semimembranosus muscles of European wild boar (Sus scrofa scrofa) meat. Can J Anim Sci.

[bib1.bib6] Borilova G, Hulankova R, Svobodova I, Jezek F, Hutarova Z, Vecerek V, Steinhauserova I (2016). The effect of storage conditions on the hygiene and sensory status of wild boar meat. Meat Sci.

[bib1.bib7] Castillo-Contreras R, Mentaberre G, Fernandez Aguilar X, Conejero C, Colom-Cadena A, Ráez-Bravo A, González-Crespo C, Espunyes J, Lavín S, López-Olvera JR (2021). Wild boar in the city: Phenotypic responses to urbanisation. Sci Total Environ.

[bib1.bib8] Coelho M, Oliveira T, Fernandes R (2013). Biochemistry of adipose tissue: an endocrine organ. Arch Med Sci.

[bib1.bib9] Chriki S, Renand G, Picard B, Micol D, Journaux L, Hocquette JF (2013). Meta-analysis of the relationships between beef tenderness and muscle characteristics. Livestock Sci.

[bib1.bib10] (2020). Concise Statistical Yearbook 2002–2019 [Internet].

[bib1.bib11] Dannenberger D, Nuernberg G, Nuernberg K, Hagemann E (2013). The effects of gender, age and region on macro- and micronutrient contents and fatty acid profiles in the muscles of roe deer and wild boar in Mecklenburg-Western Pomerania (Germany). Meat Sci.

[bib1.bib12] Dubost A, Micol D, Picard B, Lethias C, Andueza D, Bauchart D, Listrat A (2013). Structural and biochemical characteristics of bovine intramuscular connective tissue and beef quality. Meat Sci.

[bib1.bib13] Duma-Kocan P, Gil M, Stanisławczyk R, Rudy M (2019). The effect of selected methods of heat treatment on the chemical composition, colour and texture parameters of longissimus dorsi muscle of wild boars. CyTA – J Food.

[bib1.bib14] Dzierżyńska-Cybulko B, Fruziński B (1997). Dziczyzna jako źródło żywności [Wild animals as a food source].

[bib1.bib15] Eaton SA, Sethi JK (2019). Immunometabolic Links between Estrogen, Adipose Tissue and Female Reproductive Metabolism. Biology (Basel).

[bib1.bib16] Herrero J, García-Serrano A, Couto S, Ortuño VM, García-González R (2006). Diet of wild boar Sus scrofa L. and crop damage in an intensive agroecosystem. Eur J Wildl Res.

[bib1.bib17] (1995). From October 11, 1995 with
later changes [Internet].

[bib1.bib18] Janiszewski P, Daszkiewicz T (2010). Zwierzęta łowne. Zasady prawidłowego pozyskania i zagospodarowania [Game animals. Principles of proper acquisition and management].

[bib1.bib19] Kasprzyk A (2015). A comparison of chemical and physical parameters of musculus longissimus dorsi from wild boars and pigs. Annales UMCS, sec EE, Zootechnica.

[bib1.bib20] Kasprzyk A, Stasiak D, Stadnik J, Lechowski J, Stasiak A (2013). Wpływ masy tuszy dzików na wybrane cechy jakości mięsa [Influence of wild boar carcass weight on selected meat quality characteristics].

[bib1.bib21] Korzeniowski W, Bojarska U, Cierach M (1991). Wartość odżywcza mięsa dzików [The nutritional value of wild boar meat]. Med Wet.

[bib1.bib22] Korzeniowski W, Bojarska U, Cierach M (1991). Wartość rzeźna dzika [The slaughter value of a wild boar]. Medycyna Wet.

[bib1.bib23] Kujawskie Koło Łowieckie (2012). Odstrzałstrukturalny dzików [Structural harvest of wild boars].

[bib1.bib24] Ludwiczak A, Kulig D, Składanowska-Baryza J, Bykowska-Maciejewska M, Tarnawski T, Stanisz M (2019). The effect of chilled storage on the quality of meat from the feral wild boar (Sus scrofa). Ital J Anim Sci.

[bib1.bib25] Ludwiczak A, Składanowska-Baryza J, Stanisz M (2020). Effect of age and sex on the quality of offal and meat of the wild boar (Sus scrofa). Animals.

[bib1.bib26] (2020). Gatunki łowne, dzik [Game species, wild boar].

[bib1.bib27] Marsico G, Rasulo A, Dimatteo S, Tarricone S, Pinto F, Ragni M (2007). Pig, F1 (wild boar × pig) and wild boar meat quality. Ital J Anim Sci.

[bib1.bib28] Modzelewska-Kapituła M, Nogalski Z (2014). Effect of gender on collagen profile and tenderness of infraspinatus and semimembranosus muscles of Polish Holstein-Friesian x Limousine crossbred cattle. Livestock Sci.

[bib1.bib29] Modzelewska-Kapituła M, Nogalski Z, Kwiatkowska A (2014). Comparison of collagen profile and tenderness of the muscles from heifers and single-calf cows. S Afr J Anim Sci.

[bib1.bib30] Oshima I, Iwamoto H, Nakamura YN, Takayama K, Ono Y, Murakami T, Shiba N, Tabata S, Nishimura S (2009). Comparative study of the histochemical properties, collagen content andarchitecture of the skeletal muscles of wild boar crossbred pigs and commercial hybrid pigs. Meat Sci.

[bib1.bib31] Pałubicki J, Kosicki R, Twarużek M, Ałtyn I, Grajewski J (2021). Concentrations of zearalenone and its metabolites in female wild boars from woodlands and farmlands. Toxicon.

[bib1.bib32] Pedrazzoli M, Dal Bosco A, Castellini C, Ranucci D, Mattioli S, Pauselli M, Roscini V (2017). Effect of age and feeding area on meat quality of wild boars. Ital J Anim Sci.

[bib1.bib33] Polish Committee for Standardization (2000). PN-ISO 3496, Mięso i przetwory mięsne – Oznaczanie zawartości hydroksyproliny [Meat and meat products – Hydroxyproline contents determination].

[bib1.bib34] Polish Law Journal No. 61/548 (2005). Regulation No 61, position 548 of the Minister of the Environment of 22 March 1995 on detailed conditions of hunting and marking of carcasses, with later amends.

[bib1.bib35] Popczyk B (2016). Zarządzanie populacją dzika Sus scrofa w Polsce [Management of wild boar Sus scrofa population in Poland].

[bib1.bib36] Postolache AN, Lazăr R, Boişteanu PC (2010). Researches on the characterization of physical and chemical parameters of refrigerated meat from wild boar sampled from the n-e part of Romania. Lucrări Ştiinţifice, Seria Zootehnie.

[bib1.bib37] Przybylski A (2006). Dziczy fenomen [A wild phenomenon]. Łowiec Polski.

[bib1.bib38] Razmaite V, Švirmickas GJ, Šiukščius A (2012). Effect of weight, sex and hunting period on fatty acid composition of intramuscular and subcutaneous fat from wild boar. Ital J Anim Sci.

[bib1.bib39] Rede R, Pribisch V, Rehelić S (1986). Untersuchungen über die Beschaffenheit von Schlachttierkörpern und Fleisch primitiver und hochselektierter Schweinerassen
[Investigations into the condition of carcasses and meat of primitive and highly selected pig breeds]. Fleischwirtsch.

[bib1.bib40] Regulation (EC) No 853/2004 (2004). The European Parliament and of The Council of 29 April 2004 laying down specific hygiene rules for on the hygiene of foodstuffs.

[bib1.bib41] Ristić S, Żivković J, Anićić V (1987). Prilog poznawanju kvaliteta mesa divljih svinja [Contribution to the knowledge of the quality of wild boar meat]. Tehnologija Mesa.

[bib1.bib42] Russo C, Balloni S, Altomonte I, Martini M, Nuvoloni R, Cecchi F, Pedonese F, Salari F, Sant'ana Da Silva AM, Torracca B, Profumo A (2017). Fatty acid and microbiological profile of the meat (longissimus dorsi muscle) of wild boar (Sus scropha scropha) hunted in Tuscany. Ital J Anim Sci.

[bib1.bib43] Sales J, Kotrba R (2013). Meat from wild boar (Sus scrofa L.): A review. Meat Sci.

[bib1.bib44] Scillitani L, Monaco A, Toso S (2010). Do intensive drive hunts affect wild boar (Sus scrofa) spatial behaviour in Italy? Some evidences and management implications. Eur J Wildl Res.

[bib1.bib45] Skewes O, Morales R, Gonzalez F, Lui J, Hofbauer P, Paulsen P (2008). Carcass and meat quality traits of wild boar (Sus scrofa s.L.) with 2n = 36 karyotype compared to those of phenotypically similar crossbreeds (2n = 37 and 2n = 38) raised under same farming conditions. 1. Carcass quantity and meat dressing. Meat Sci.

[bib1.bib46] Standard BN-84/924/-10 (1984). Mięso z dziczyzny. Tusze, półtusze, ćwierćtusze i elementy z dziczyzny mrożone [Game meat. Frozen carcasses, half-carcasses, quarters and game cuts].

[bib1.bib47] Strazdina V, Jemeljanovs A, Sterna V, Ikauniece D (2013). Nutrition value of deer, wild boar and beaver meat hunted in Latvia.

[bib1.bib48] Strazdina V, Jemeljanovs A, Sterna V, Ikauniece D (2015). Nutritional characteristics of wild boar meat hunted in Latvia. Conference Proceedings.

[bib1.bib49] Szmańko T, Górecka J, Korzeniowska M, Malicki A, Eeremenko E (2007). Comparison of chosen quality parameters of meat from wild boar and domestic pigs. Pol J Food Nutr Sci.

[bib1.bib50] Tatum JD (2011). Animal age, physiological maturity, and associated effects on beef tenderness.

[bib1.bib51] Tesarova S, Jezek F, Hulankova R, Plhal R, Drimaj J, Steinhauserova I, Borilova G (2018). The individual effect of different production systems, age and sex on the chemical composition of wild boar meat. Acta Vet Brno.

[bib1.bib52] Tropiło J, Kiszczak L (2008). Badanie i ocena sanitarno-weterynaryjna zwierząt łownych i dziczyzny [Sanitary and veterinary inspection and evaluation of game animals].

[bib1.bib53] Żmijewski T, Korzeniowski W (2001). Technological properties of wild boars meat. Electron J Pol Agri Univ, Food Sci Technol.

[bib1.bib54] Żmijewski T, Korzeniowski W (2000). Tissue composition of wild boars carcasses. Electron J Pol Agri Univ, Food Sci Technol.

[bib1.bib55] Żmijewski T, Cierach M, Kwiatkowska A (2007). Wartość użytkowa tusz zwierząt łownych [Carcass value of game animals]. Rocz Inst Przem Mięs Tłuszcz.

[bib1.bib56] Żochowska J, Lachowicz K, Gajowiecki L, Sobczak M, Żych A, Kotowicz M (2004). Wydajność łowna, udziałelementów zasadniczych oraz wyciek cieplny i pH mięsa dzików o różnej masie [The dressing percentage and percentage of primary cuts in carcasses, thermal drip losses and pH of wild boars meat of different weight]. Folia Pomeranae Universitatis Technologiae Stetinensis.

[bib1.bib57] Żochowska J, Lachowicz K, Gajowiecki L, Sobczak M, Kotowicz M, Żych A (2005). Effects of carcass weight and muscle on texture, structure and myofibre characteristics of wild boar meat. Meat Sci.

[bib1.bib58] Żochowska-Kujawska J, Lachowicz K, Sobczak M, Bienkiewicz G (2010). Utility for production of massaged products of selected wild boar muscles originating from wetlands and an arable area. Meat Sci.

[bib1.bib59] Żochowska-Kujawska J, Lachowicz K, Sobczak M, Nitek L (2010). Wydajność łowna i udziałelementów zasadniczych w tuszach dzików z zależności od sezonu i miejsca odstrzału oraz płci [Dressing percentage and the percentage of prime cuts in the carcasses of wild boars depending on the season and region of shooting and sex]. Med Wet.

